# Real-world clinical characteristics and outcomes of patients with locally advanced, unresectable esophageal/gastroesophageal junction cancer treated with definitive chemoradiotherapy in the United States community oncology setting

**DOI:** 10.3389/fonc.2025.1679386

**Published:** 2026-01-12

**Authors:** Adriana Valderrama, Karthik Ramakrishnan, Lisa Herms, Helen Latimer, Junxin Shi, Gregory Patton, Sonal Bordia, Sujatha Nallapareddy

**Affiliations:** 1 Merck & Co., Inc., Rahway, NJ, United States; 2 Real World Research, Ontada, Boston, MA, United States; 3 Rocky Mountain Cancer Centers, The US Oncology Network, Aurora, CO, United States

**Keywords:** locally advanced esophageal cancer, gastric or gastroesophageal junction cancer, survival analysis, treatment patterns, recurrent or progressive disease, definitive chemoradiotherapy, real-world data, patient outcomes

## Abstract

**Introduction:**

There are limited real-world data describing treatment patterns and clinical outcomes for patients with locally advanced, unresectable esophageal and gastroesophageal junction cancer (EC/GEJC) treated with definitive chemoradiotherapy (dCRT).

**Methods:**

This retrospective study included patients diagnosed with EC/GEJC who initiated dCRT between January 1, 2015, and June 30, 2021, within a large network of community oncology centers. Data from the electronic health record database were used. Demographic and clinical characteristics were evaluated in patients overall and stratified by disease recurrence status. Treatment characteristics, including index chemotherapy regimen and radiation dose, were assessed descriptively. Real-world time on treatment (rwTOT), real-world overall survival (rwOS), real-world event-free survival (rwEFS), and real-world recurrence-free survival (rwRFS) were assessed using Kaplan-Meier methods. Correlation between rwEFS and rwOS was estimated using Kendall-Tau’s correlation coefficient.

**Results:**

A total of 17,427 patients were identified with a diagnosis of EC/GEJC. After meeting all eligibility requirements, 300 patients who initiated dCRT were included in the study population, with 37.3% of patients experiencing recurrence during follow-up; median follow-up time was 10.5 (interquartile range: 4.0, 21.0) months overall, with median follow-up of 14.1 months among patients with recurrence and 6.4 months among patients without recurrence. Carboplatin + paclitaxel (86.0%) was the most common chemotherapy with concurrent radiation treatment. Nearly half (46.3%) of the cohort received radiation dosing between 50-50.4 Gray. Median rwEFS was 8.9 (95% confidence interval [CI]: 7.7, 10.6) months, median rwRFS was 14.0 months, and median rwOS was 18.1 (95% CI: 13.3, 21.8) months. Landmark OS at 6 months for patients with recurrence was 7.1 (95% CI: 2.9, 13.2) months and 21.0 (95% CI: 17.6, 44.8) months for patients without a recurrence. Similarly, landmark OS at 12 months in the recurrence subgroup was 8.5 (95% CI: 6.8, 12.0) months and 41.5 (95% CI: 38.8, not reported [NR]) months in the non-recurrence subgroup. Furthermore, rwEFS and rwOS had a strong correlation (r = 0.8; 95% CI: 0.8, 0.9), indicating a delay in recurrence was associated with improved survival.

**Conclusion:**

The results of this analysis emphasize an unmet need for more effective therapies for EC/GEJC patients to prevent disease recurrence and improve outcomes.

## 1 Introduction

As of 2022, esophageal cancer was the eleventh most diagnosed cancer and the seventh leading cause of cancer-related deaths worldwide (1). The number of new cases is projected to increase by 31.4% in 2030 and 63.5% in 2040 (2). In the United States (US), esophageal cancer currently accounts for 1% of all cancers diagnosed, with an estimated 22,370 new cases and 16,250 related deaths anticipated in 2025 (3).

The two primary types of esophageal cancer are squamous cell carcinoma and adenocarcinoma (4). In the US and most Western countries, adenocarcinoma is the predominant subtype, typically occurring in the distal esophagus and gastroesophageal junction (GEJ) (5). Key risk factors for developing esophageal adenocarcinoma include chronic gastroesophageal reflux disease, obesity, and smoking (4, 6). Most esophageal cancers are diagnosed at advanced stages, resulting in a poor prognosis and low survival rates (7). Despite recent treatment advancements, the overall 5-year survival rate remains around 10%, with post-esophagectomy survival rates ranging from 15% to 40% (8).

Locally advanced esophageal and GEJ cancer (EC/GEJC) can be managed through various treatment options, including endoscopic resection, surgery (esophagogastrostomy), and neoadjuvant and adjuvant therapies (9). Surgery is generally recommended for patients with resectable disease before metastasis occurs, and definitive chemoradiotherapy (dCRT) is recommended for those who are ineligible for surgery (e.g., patients with unresectable tumors) (10). Clinical trials, such as the Radiation Therapy Oncology Group (RTOG) 85–01 trial, have demonstrated improved survival outcomes for patients receiving dCRT compared to chemotherapy or radiation alone (11). However, the eligibility criteria for these trials were often highly restrictive, and there have been few published studies evaluating real-world patient outcomes. Additionally, despite advancements, treatment options are still limited, and patients with locally advanced unresectable EC/GEJC often face relapse or disease progression, with a 5-year overall survival (OS) rate of approximately 29% (12). There is also limited real-world research on this subset of patients who experience recurrence following dCRT treatment.

To address these gaps, this study aimed to evaluate the demographics, clinical characteristics, treatment patterns, and clinical outcomes of patients diagnosed with locally advanced, unresectable EC/GEJC who were treated with dCRT at community-based oncology centers in the US.

## 2 Materials and methods

### 2.1 Study design

This retrospective observational cohort study included adult patients (≥ 18 years of age) diagnosed with locally advanced, unresectable EC/GEJC who initiated dCRT (i.e., concurrent chemotherapy and radiation administered with curative intent, without prior surgery or systemic therapy), between January 1, 2015, and June 30, 2021. Patients were followed longitudinally until the last patient record, date of death, or the end of the study period (December 31, 2022), whichever occurred first. The index date was defined as the initiation of dCRT. Eligibility required patients to have had at least two clinic visits within The US Oncology Network after diagnosis and during the study period. Patients were excluded if they had documentation of surgery for EC/GEJC or systemic therapy prior to the start of dCRT (as confirmed through chart abstraction), enrollment in interventional clinical trials during the study period, or any systemic treatment for another primary cancer during the study period.

### 2.2 Data source

Data were collected from practices within The US Oncology Network that utilize the iKnowMed™ (iKM) electronic health record (EHR). The US Oncology Network is affiliated with approximately 2,500 physicians across more than 600 sites of care in 31 states in the US, treating over 1.4 million cancer patients annually (13). iKM is an oncology-specific EHR system that captures outpatient practice encounter histories for patients receiving community-based care. Structured data were obtained via programmatic database extraction and supplemented with unstructured data captured through chart abstraction. Death records were aggregated from structured and unstructured fields and supplemented with repositories of claims data, obituary records, and the Social Security Administration’s Limited Access Death Master File.

### 2.3 Baseline characteristics

Patient demographics, including age on index, as well as gender, race, and tobacco use in baseline (within 30 days of index), were assessed descriptively and stratified by recurrence status. The following clinical characteristics were similarly assessed: tumor stage at diagnosis, time from initial diagnosis to the start of the dCRT treatment regimen (i.e., index date), Eastern Cooperative Oncology Group (ECOG) performance status, histology at diagnosis, and tumor location (i.e., either EC or GEJC). Follow-up time was also assessed overall and by recurrence status stratifications.

### 2.4 Outcomes

Treatment patterns outcomes including type and regimen name of index treatment, total dose of radiation, patient disposition, and real-world time-on-treatment (rwTOT) were assessed descriptively for the overall population. The primary outcome of this analysis was real-world overall survival (rwOS), which was assessed overall and stratified based on recurrence status. The ‘recurrence’ subgroup included patients who experienced locoregional progression (LRP) or distant metastatic recurrence or progression (DM) as documented by a physician in the patient’s progress notes at any time during the follow-up period. Patients who did not experience any of these events were classified into the ‘no recurrence’ subgroup. Additional outcomes included real-world recurrence-free survival (rwRFS) and real-world event-free survival (rwEFS).

For all outcomes, patients who did not experience a qualifying event within the study period were censored at the last contact date on or before the study end date, or the date of death (for rwRFS), whichever occurred first. rwTOT was defined as the interval between the index date and the discontinuation of the index treatment for any cause, including death. rwRFS was defined as the interval between the index date and the first occurrence of LRP or DM. rwEFS was defined as the interval between the index date and the first occurrence of LRP, DM, or death. rwOS was defined as the interval between the index date and the date of death. Additionally, rwOS was assessed from 6, 12, and 18-month landmark points to the date of death. Patients who died or were censored prior to the respective landmark time point were excluded from the landmark analysis for that specific time point. Patients were stratified by recurrence status as of each landmark point.

### 2.5 Statistical analysis

Baseline characteristics were assessed descriptively. Categorical variables were reported as frequency and percentage. Continuous variables were reported as median values with corresponding interquartile range (IQR). If missing observations were present, the number and percentage of missing values were also reported. Time-to-event outcomes (i.e., rwOS, rwTOT, rwRFS, and rwEFS) were analyzed using the Kaplan-Meier method with median time and 95% confidence intervals (CIs) reported. For rwOS, rwRFS, and rwEFS, survival probability at each month-based interval was also included. Stratifications of categorical clinical characteristics (e.g., ECOG, histology, stage, and tumor location) were presented for rwOS and rwEFS, with log-rank p-values. Baseline characteristics associated with rwOS and rwEFS were identified separately for each outcome using multivariable Cox proportional hazards modeling. Independent variables were selected based on previous literature reporting statistically significant factors in similar studies (e.g., age, sex, tumor location, tumor histology, dCRT treatment regimen on index) (14). Missing data was included in the model within the category of ‘Not Documented’ (if present) to reflect real-world documentation practices. Hazard ratios (HRs) and associated 95% CIs were calculated for each covariate.

The relationship between rwOS and recurrence was examined using the Cox proportional hazards model at 6, 12, and 18 months. Each model reflected a subgroup of patients who had survived up to each timepoint. rwOS served as the dependent variable, with patient recurrence status at the landmark time points as the primary variable of interest. Additional independent variables were consistent with those used in the rwOS and rwEFS models. Furthermore, the relationship between rwOS and rwEFS was evaluated using Kendall’s Tau correlation coefficient. For this analysis, rwEFS served as the independent variable and rwOS as the outcome of interest, with 95% CI calculated for the estimated correlation coefficient.

## 3 Results

### 3.1 Study attrition

**Figure 1:**
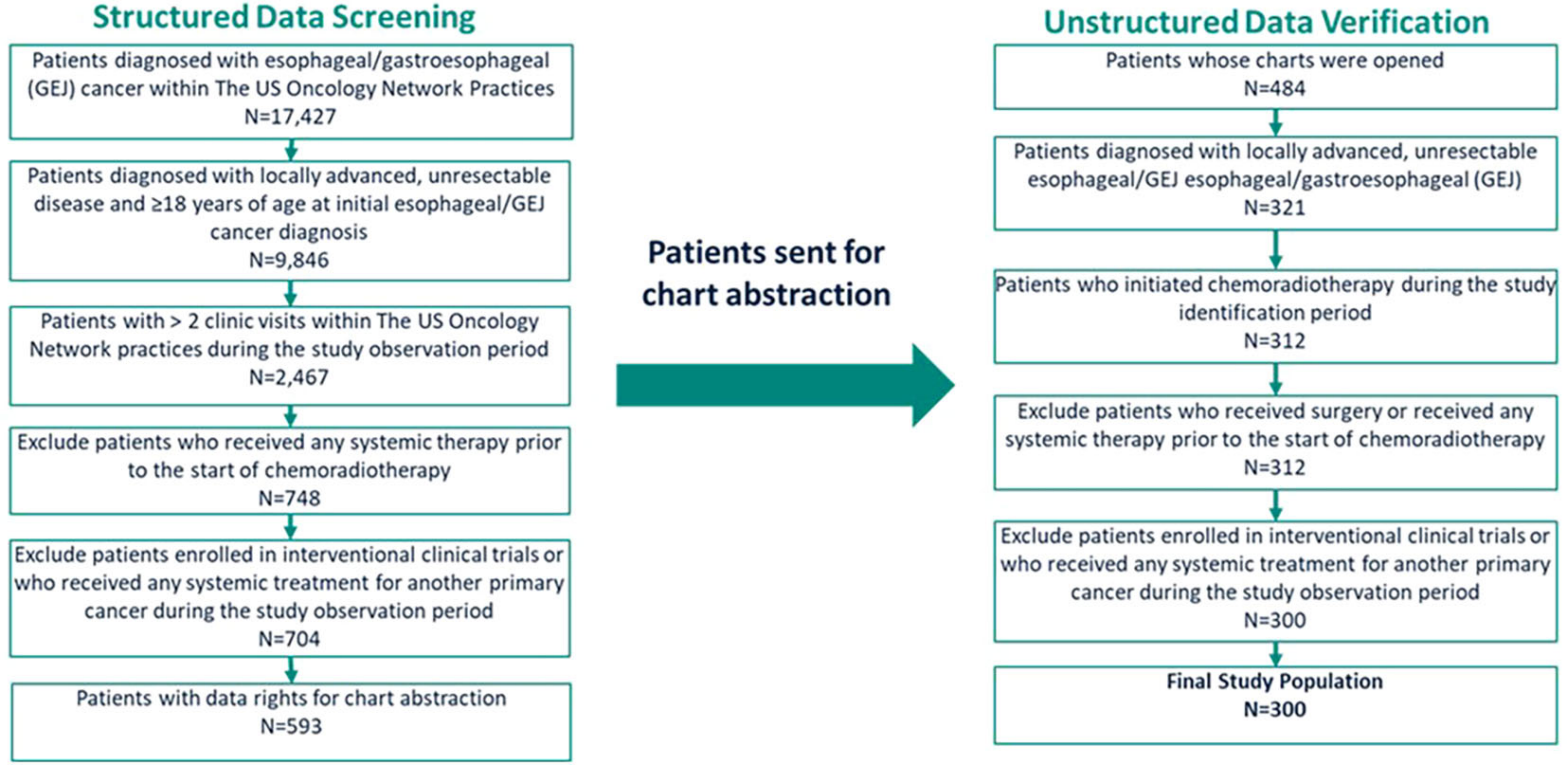
Study attrition flow diagram.

The final study cohort attrition is presented in **Figure 1**. Initially, 17,427 patients with a diagnosis of EC/GEJC at any stage were identified within The US Oncology Network based on the structured data. After applying additional eligibility criteria, to only include adult patients with locally advanced or unresectable disease treated with dCRT without prior surgery or treatment with systemic therapy during the study identification period, 529 patients were selected for chart abstraction. Eligibility was reconfirmed using unstructured data and during this process, 163 patients were excluded because they did not have locally advanced, unresectable EC/GEJC. An additional 21 patients were excluded for not initiating dCRT during the identification period, receiving surgery or systemic treatment prior to the start of dCRT, being enrolled in interventional clinical trials, or receiving systemic treatment for another primary cancer during the study period. The final analysis included 300 patients, of whom 112 (37.3%) experienced recurrence and 188 (62.7%) did not. Median follow-up time from the index date for the overall study population was 10.5 months (IQR: 4.0, 21.0; **Table 1**). Patients who experienced recurrence had a median follow-up of 14.1 months (IQR: 8.8, 21.1; **Table 1**), while those who did not experience recurrence had a median follow-up of 6.4 months (IQR: 2.7, 20.3; **Table 1**).

**Table 1:** Baseline patient characteristics.

Variable	Overall N=300	Recurrence N=112	No recurrence N=188
Median (IQR) Age at Index	73 (64,80)	72 (62,80)	73 (66,80)
Age group at index, n (%)			
<65 Years	78 (26.0%)	36 (32.1%)	42 (22.3%)
≥65 Years	222 (74.0%)	76 (67.9%)	146 (77.7%)
Gender, n (%)			
Female	77 (25.7%)	26 (23.2%)	51 (27.1%)
Male	223 (74.3%)	86 (76.8%)	137 (72.9%)
Race, n (%)			
Documented race	248 (82.7%)	98 (87.5%)	150 (79.8%)
Black/African American	19 (7.7%)	<10	14 (9.3%)
White/Caucasian	221 (89.1%)	90 (91.8%)	131 (87.3%)
Other	<10	<10	<10
Not documented	52 (17.3%)	14 (12.5%)	38 (20.2%)
Tobacco use at baseline, n (%)			
No History of Tobacco Use	64 (21.3%)	21 (18.8%)	34 (18.1%)
Current Tobacco Use	55 (18.3%)	60 (53.6%)	93 (49.5%)
Former Tobacco Use	153 (51.0%)	17 (15.2%)	47 (25.0%)
Not documented	28 (9.3%)	14 (12.5%)	14 (7.4%)
Median (IQR) Patient Follow-Up Time (months)	10.5 (4.0,21.0)	14.1 (8.8,21.1)	6.4 (2.7,20.3)
Stage at initial diagnosis, n (%)^1^			
Stage II	104 (34.7%)	38 (33.9%)	66 (35.1%)
Stage III	167 (55.7%)	65 (58.0%)	102 (54.3%)
Stage IVA	16 (5.3%)	<10	11 (5.9%)
Not documented	13 (4.3%)	<10	<10
Median (IQR) Time from Initial Diagnosis to Index Treatment (weeks)	5.7 (4.0,7.9)	5.3 (4.0,7.5)	5.9 (3.9,8.0)
ECOG at baseline, n (%)			
Documented ECOG	251 (83.7%)	93 (83.0%)	158 (84.0%)
0-1	190 (75.7%)	71 (76.3%)	119 (75.3%)
≥2	61 (24.3%)	22 (23.7%)	39 (24.7%)
Not documented	49 (16.3%)	19 (17.0%)	30 (16.0%)
Histology at diagnosis, n (%)^2^			
Adenocarcinoma	159 (53.0%)	64 (57.1%)	95 (50.5%)
Squamous Cell	116 (38.7%)	37 (33.0%)	79 (42.0%)
Other	25 (8.3%)	11 (9.8%)	14 (7.4%)
Initial tumor location at diagnosis, n (%)			
Esophageal	212 (70.7%)	75 (67.0%)	137 (72.9%)
Gastroesophageal Junction	88 (29.3%)	37 (33.0%)	51 (27.1%)

IQR, interquartile range; ECOG, Eastern Cooperative Oncology Group

Index is the initiation of LOT1 chemoradiotherapy. Baseline is the closest value within 30 days (before or after) of index. To comply with Ontada’s data reporting standards, categorical variables with less than 10 patients were masked to protect patient privacy and prevent re-identification.

^1^Those without a documented stage had a documented TNM that met the inclusion criteria.

^2^Other histology include: adenosquamous, other, signet-ring cell carcinoma, small cell carcinoma.

### 3.2 Demographic and clinical characteristics

Demographic and clinical characteristics are reported in **Table 1**. The median age at index for the overall population was 73 years (IQR: 64, 80), with a similar median age observed in both the recurrence and no recurrence subgroups (72 years [IQR: 62, 80] and 73 years [IQR: 66, 80], respectively). Overall, 74.0% (n=222) of the study participants were over 65 years.

In addition to age, other demographics of the study population were also comparable between patients with and without recurrence. Overall, 74.3% (n=223) of the study population was male with 76.8% (n=86) in the recurrence subgroup and 72.9% (n=137) in the no recurrence group being male. The majority of the overall study population (89.1%, n=221) were White/Caucasian, 7.7% (n=19) were Black/African American, and fewer than 10 patients had other racial categories. At baseline, 51.0% (n=153) of patients had a history of former tobacco use, 18.3% (n=55) were current tobacco users, and 21.3% (n=64) had no history of tobacco use. At diagnosis, 34.7% (n=104) of patients were stage II, 55.7% (n=167) were stage III, 5.3% (n=16) were stage IVA, and 4.3% (n=13) had an undocumented stage. Patients with an undocumented stage had other tumor, node, or metastasis information confirming locally advanced, unresectable status.

For ECOG performance status at baseline, among those with a documented ECOG (n=251), 75.7% (n=190) had an ECOG score of 0-1, and 24.3% (n=61) had a score of 2 or higher. At diagnosis, 53.0% (n=159) of patients had adenocarcinoma, 38.7% (n=116) had squamous cell carcinoma, and 8.3% (n=25) had other histologies (i.e., adenosquamous, signet-ring cell carcinoma, small cell carcinoma, and otherwise not specified). Most patients (70.7%, n=212) had esophageal tumors, while 29.3% (n=88) of patients had tumors at the GEJ. The median time from initial diagnosis to index treatment was 5.7 (IQR: 4.0, 7.9) weeks overall. In the recurrence stratifications, the time from initial diagnosis to index treatment was similar: 5.3 (4.0, 7.5) weeks for patients with recurrence and 5.9 (3.9, 8.0) weeks for those without.

### 3.3 Treatment patterns

The most common index regimens included carboplatin + paclitaxel (86.0%, n=258), followed by cisplatin + fluorouracil (9.0%, n=27), and other regimens (5.0%, n=15; **Table 2**). Nearly all patients (99.7%, n=299) received concurrent rather than sequential dCRT as their index treatment. The distribution of radiation doses among patients varied, with 17.3% (n=52) receiving less than 50 Gray (Gy), 46.3% (n=139) receiving between 50 and 50.4 Gy, 17.7% (n=53) receiving more than 50.4 Gy.

**Table 2:** Treatment patterns.

Variable	Overall N=300
Index treatment Regimens, n (%)	
Carboplatin + paclitaxel	258 (86.0%)
Cisplatin + fluorouracil	27 (9.0%)
Other regimens **^1^**	15 (5.0%)
Type of index treatment, n (%)	
Concurrent	299 (99.7%)
Sequential	<10
Total dose of radiation (Gy), n (%)	
<50	52 (17.3%)
50-50.4	139 (46.3%)
>50.4	53 (17.7%)
Not documented	56 (18.7%)
Patient disposition following index, n (%)	
Started second-line (LOT2) treatment	62 (20.7%)
Died without starting LOT2 treatment	117 (39.0%)
Did not die and did not start LOT2 treatment	121 (40.3%)
Median (95% Confidence Interval) Real-world Time-on-Treatment	1.4 (1.3,1.4)

LOT, line of therapy

comply with Ontada’s data reporting standards, categorical variables with less than 10 patients were masked to protect patient privacy and prevent re-identification.

^1^ Other regimens include cisplatin + paclitaxel + trastuzumab, capecitabine + cisplatin, carboplatin, carboplatin + etoposide, cisplatin, cisplatin + docetaxel + paclitaxel, cisplatin + fluorouracil + leucovorin, cisplatin + paclitaxel, fluorouracil, and paclitaxel.

**Figure 2:**
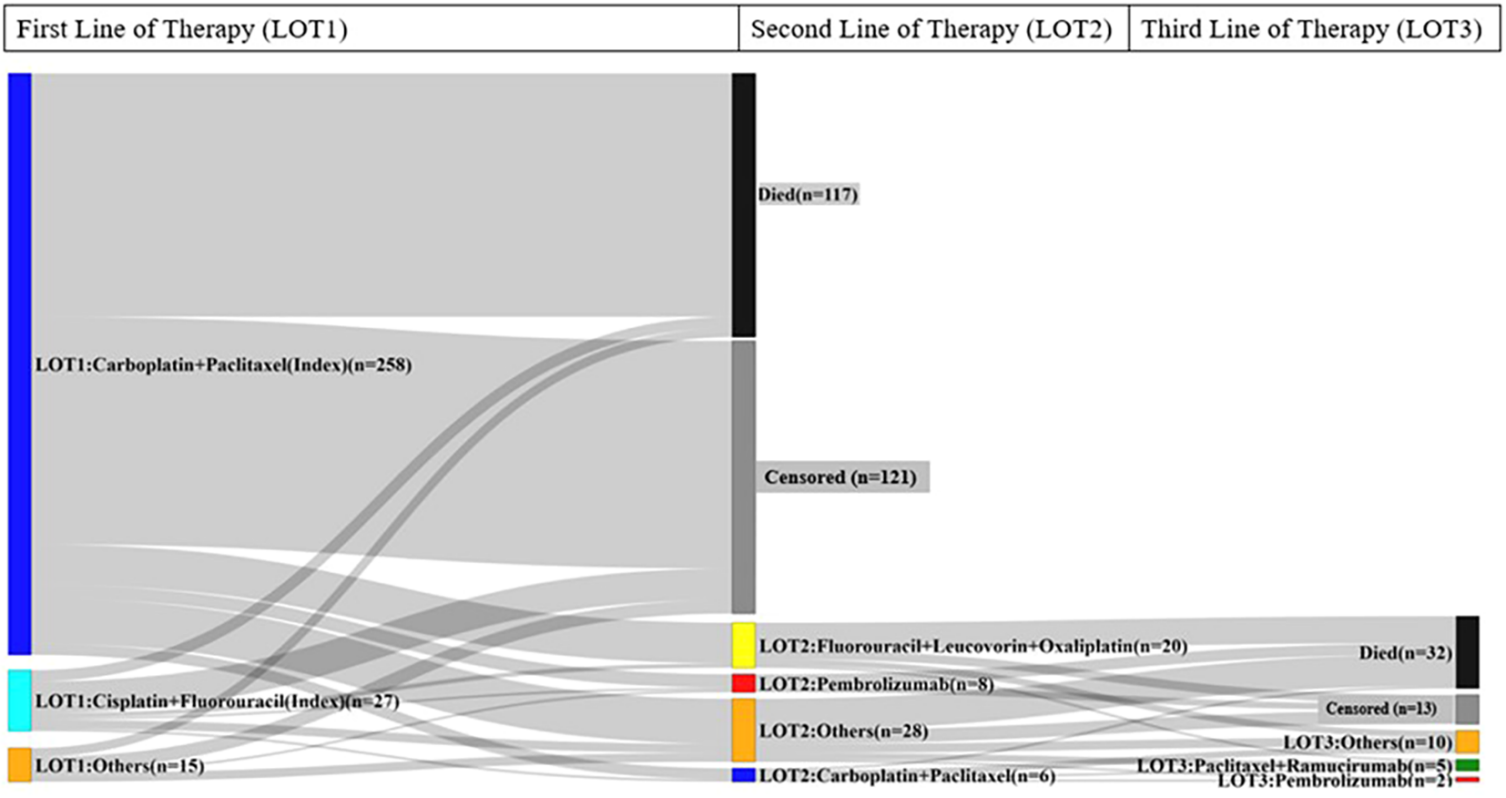
Sankey diagram of treatment sequences from the index treatment to the third-line of therapy.

The median rwTOT was 1.4 months (95% CI: 1.3, 1.4; **Table 2**). Following initiation of first-line (LOT1) index treatment, 20.7% (n=62) of patients initiated second-line (LOT2) treatment, 39.0% (n=117) died without commencing LOT2 treatment, and 40.3% (n=121) neither died nor started LOT2 treatment. Of the 62 patients who received LOT2 treatment, 27.4% (n=17) proceeded to third-line (LOT3) treatment (**Figure 2**).

### 3.4 Clinical outcomes

Results for overall survival outcomes (i.e., rwOS, rwRFS, and rwEFS) are included in **Table 3**.

**Table 3:** Clinical outcomes from index for the overall population.

Variable	rwOS	rwEFS	rwRFS
Events, N (%)	161 (53.7%)	198 (66.0%)	112 (37.3%)
Median (95% CI), Months	18.1 (13.3, 21.8)	8.9 (7.7, 10.6)	14.0 (11.2,29.1)
Survival Probability			
6 months (95% CI)	76.7% (71.3%, 81.3%)	65.9% (60.0%,71.2%)	81.4% (75.6%, 85.9%)
12 months (95% CI)	59.9% (53.5%,65.6%)	40.3% (34.3%, 46.3%)	54.7% (47.3%, 61.5%)
24 months (95% CI)	39.8% (33.2%, 46.4%)	26.9% (21.3%,32.8%)	43.0% (35.3%, 50.5%)
36 months (95% CI)	29.0% (22.3%,35.9%)	20.7% (15.3%, 26.7%)	37.5% (29.2%, 45.8%)
48 months (95% CI)	29.0% (22.3%,35.9%)	18.6% (13.2%, 24.8%)	33.7% (24.8%, 42.8%)
60 months (95% CI)	21.5% (13.3%, 30.9%)	16.5% (10.7%, 23.5%)	33.7% (24.8%,42.8%)

CI, confidence interval; rwEFS, real-world event-free survival; rwOS, real-world overall survival; rwRFS, real-world recurrence-free survival

#### 3.4.1 rwRFS and rwEFS

**Figure 3:**
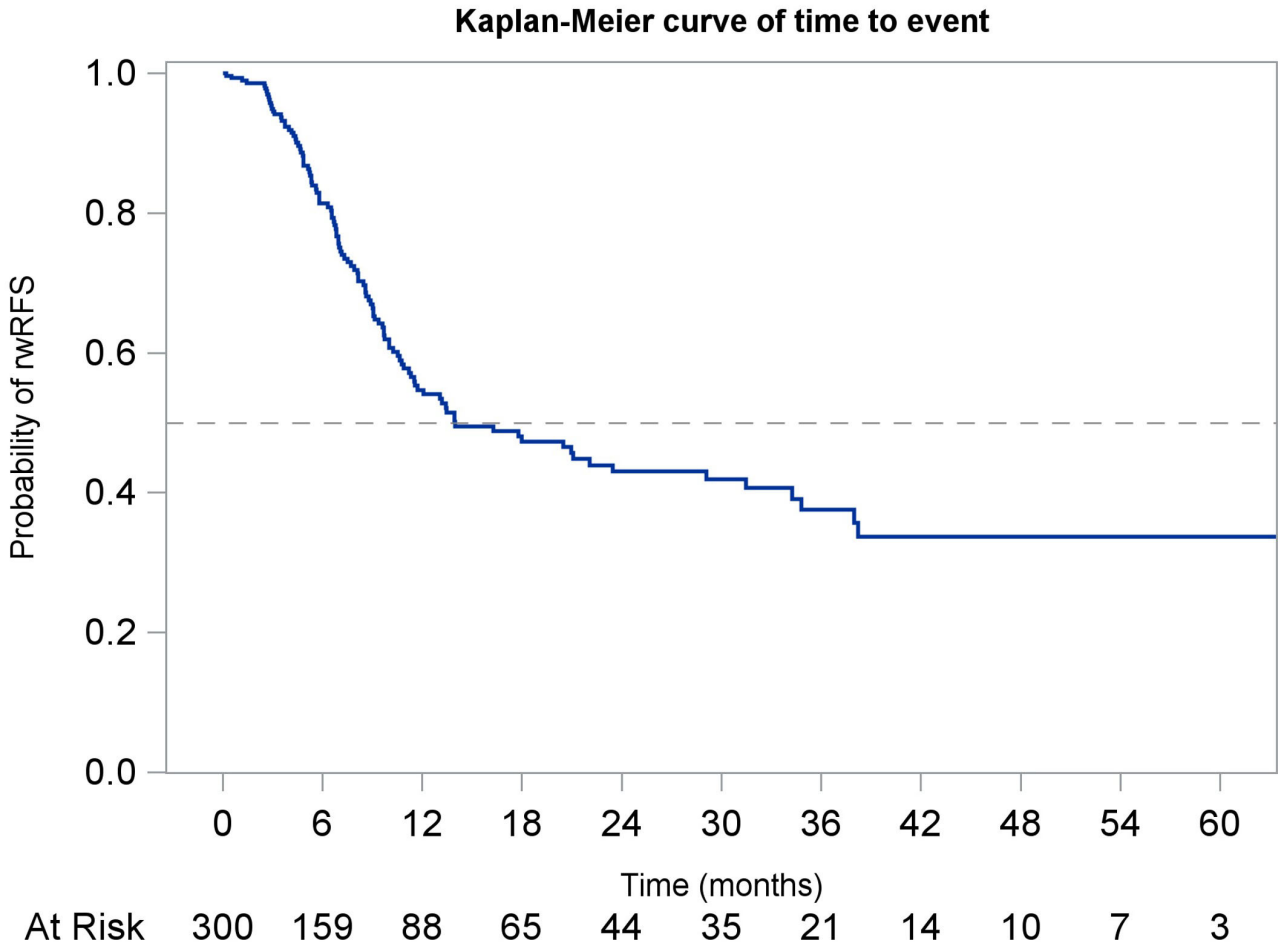
Kaplan-Meier curve of real-world recurrence-free survival (rwRFS) for the overall study population starting from index.

**Figure 4:**
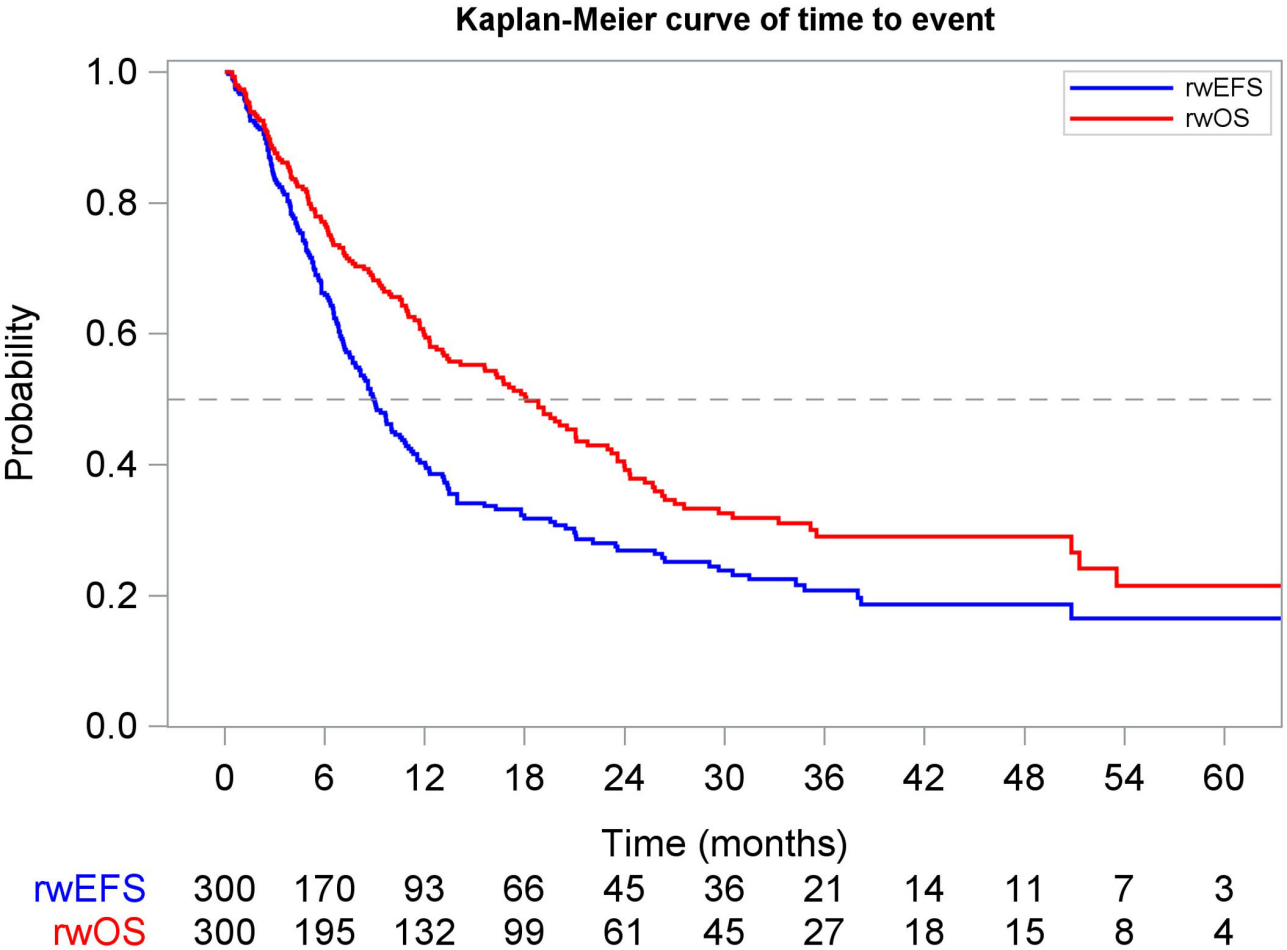
Kaplan-Meier curve of real-world overall survival (rwOS) and real-world event-free survival (rwEFS) for the overall study population starting from index.

The median rwRFS was 14.0 months (95% CI: 11.2, 29.1; **Figure 3**), and the median rwEFS was 8.9 months (95% CI: 7.7, 10.6; **Figure 4**). Baseline stratifications for rwEFS are included in **Table 4**. The rwEFS varied based on ECOG scores, with those scoring 0–1 having a median rwEFS of 9.6 months (95% CI: 7.5, 11.5), those scoring 2+ having a median rwEFS of 7.8 months (95% CI: 4.9, 11.7), and those with undocumented scores having a median rwEFS of 9.7 months (95% CI: 6.6, 13.1; p = 0.2663). The rwEFS also differed by histology, with adenocarcinoma patients having a median rwEFS of 8.6 months (95% CI: 6.9, 10.3), squamous cell patients 11.5 months (95% CI: 8.4, 18.0), and others 6.5 months (95% CI: 5.2, 10.6; p = 0.0156). Patients with stage II disease had the longest median rwEFS of 11.2 months (95% CI: 8.4, 18.0), compared to stage IVA patients at 10.0 months (95% CI: 3.5, not reported [NR]), and stage III patients at 8.1 months (95% CI: 6.5, 9.4) (p = 0.1388). Stratified analysis by tumor location showed median rwEFS of 9.1 months (95% CI: 7.7, 11.6) for esophageal tumors and 8.3 months (95% CI: 6.8, 10.9) for GEJ tumors (p = 0.227).

**Table 4:** Clinical outcomes from index for the overall population, stratified by baseline characteristics.

Baseline stratifications	rwEFS			rwOS		
	Median (95% CI), months	Odds ratio (95% CI)	P-value	Median (95% CI), months	Odds ratio (95% CI)	P-value
ECOG (0-1), n=190	9.6 (7.5, 11.5)	(reference)	–	19.2 (13.1, 24.3)	(reference)	–
ECOG (2+), n=61	7.8 (4.9, 11.7)	1.3 (0.9, 1.8)	0.2260	11.9 (7.8, 35.5)	1.1 (0.7, 1.8)	0.5258
ECOG (undocumented), n=49	9.7 (6.6, 13.1)	1.4 (1.0, 1.9)	0.0686	18.8 (11.0, 23.6)	1.4 (0.9, 1.9)	0.0964
Histology (Squamous Cell), n=116	11.5 (8.4, 18.0)	(reference)	–	19.8 (14.2, 50.8)	(reference)	–
Histology (Adenocarcinoma), n=159	8.6 (6.9, 10.3)	1.7 (1.2, 2.4)	0.0049	16.7(11.7, 21.1)	1.6 (1.1, 2.4)	0.0134
Histology (other)^1^, n=25	6.5 (5.2, 10.6)	1.8 (1.1, 3.0)	0.0260	18.0 (6.0, 25.2)	1.8 (1.0, 3.3)	0.0476
Tumor stage (II)^2^, n=104	11.2 (8.4, 18.0)	(reference)	–	24.0 (18.8, 33.2)	(reference)	–
Tumor stage (III)^2^, n=167	8.1 (6.5, 9.4)	1.5 (1.1, 2.0)	0.0186	13.1 (10.9, 18.8)	1.6 (1.1, 2.2)	0.0154
Tumor stage (IVA)^2^, n=16	10.0 (3.5, NR)	1.0 (0.5, 2.3)	0.9232	NR (4.4, NR)	1.2 (0.5, 3.0)	0.6267
Tumor location (esophageal), n=212	9.1 (7.7, 11.6)	(reference)	–	18.8 (13.1, 23.2)	(reference)	–
Tumor location (GEJ), n=88	8.3 (6.8, 10.9)	1.0 (0.7, 1.5)	0.8123	17.1 (11.9, 25.2)	0.9 (0.6, 1.4)	0.6543

CI, confidence interval; ECOG, Eastern Cooperative Oncology Group; GEJ, gastroesophageal junction; NR, not reached; rwEFS, real-world event-free survival; rwOS, real-world overall survival

^1^Other histology include: adenosquamous, other, signet-ring cell carcinoma, small cell carcinoma.

^2^There were 13 patients without a documented stage who met eligibility criteria. Results for these patients are not included in this table.

**Figure 5:**
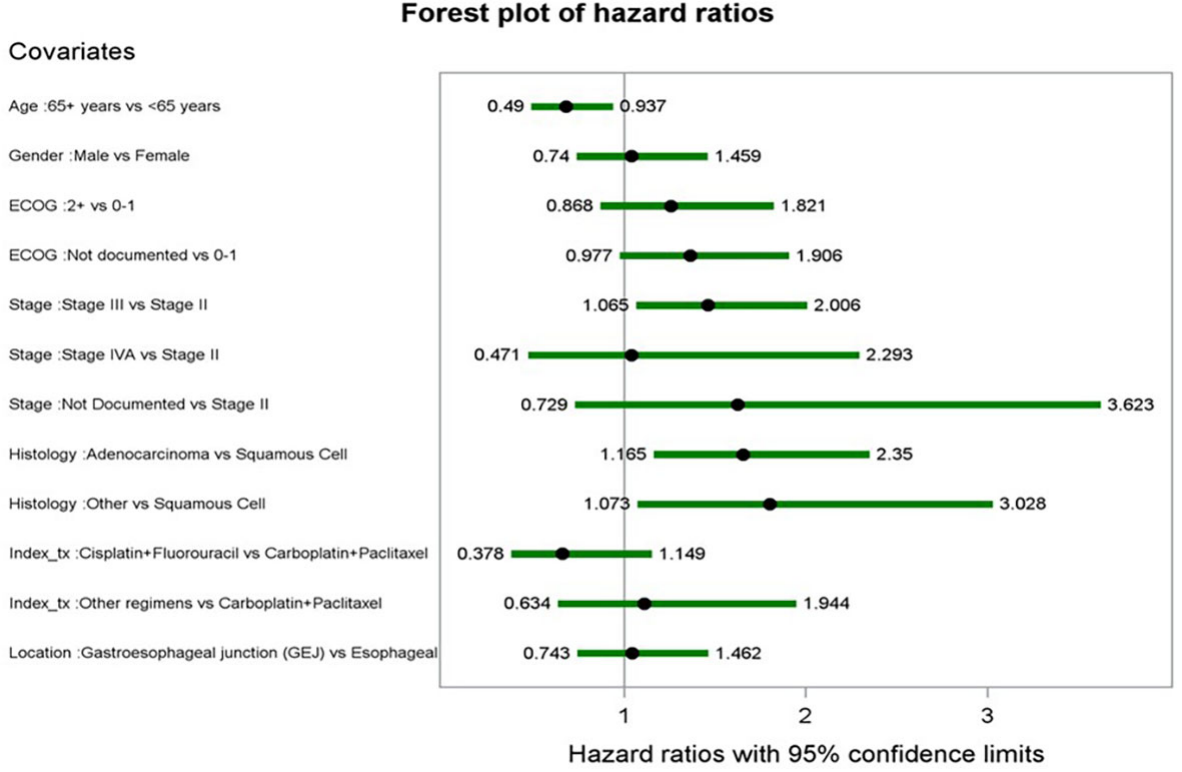
Forest plot of hazard ratios from multivariate cox proportional hazards model for rwEFS.

Covariates and their corresponding hazard ratios (HR) from the multivariable Cox proportional hazard model for rwEFS are shown in **Figure 5**. Older age at index (65+ years vs. ≤ 65 years) was associated with a lower risk of an rwEFS event, with an HR of 0.678 (p < 0.0184). Patients with stage III at diagnosis had higher risk of an rwEFS event compared to stage II patients (HR: 1.462, p = 0.0186), as did patients with adenocarcinoma (vs. squamous cell) (HR: 1.655, p = 0.0049) and other histology types (vs. squamous cell) (HR: 1.803, p = 0.0260). Factors included in the final model but not statistically significant (p < 0.05) were sex, ECOG performance status at baseline, index dCRT regimen, and tumor location.

#### 3.4.2 rwOS

The median rwOS for the study population was 18.1 months (95% CI: 13.3, 21.8) (**Figure 4**). Baseline stratifications for rwOS are included in **Table 4**. Median rwOS also differed by ECOG performance status, with those patients scoring 0–1 having a median rwOS of 19.2 months (95% CI: 13.1, 24.3), those scoring 2+ having a median rwOS of 11.9 months (95% CI: 7.8, 35.5), and those with undocumented scores having a median rwOS of 18.8 months (95% CI: 11.0, 23.6) (p = 0.4459). Patients with adenocarcinoma had a median rwOS of 16.7 months (95% CI: 11.7, 21.1), squamous cell patients 19.8 months (95% CI: 14.2, 50.8), and others 18.0 months (95% CI: 6.0, 25.2) (p = 0.112). Stage II patients had a median rwOS of 24.0 months (95% CI: 18.8, 33.2), and stage III patients had a median rwOS of 13.1 months (95% CI: 10.9, 18.8). Analysis by tumor location showed median rwOS of 18.8 months (95% CI: 13.1, 23.2) for esophageal tumors and 17.1 months (95% CI: 11.9, 25.2) for GEJ tumors (p = 0.8779).

**Figure 6:**
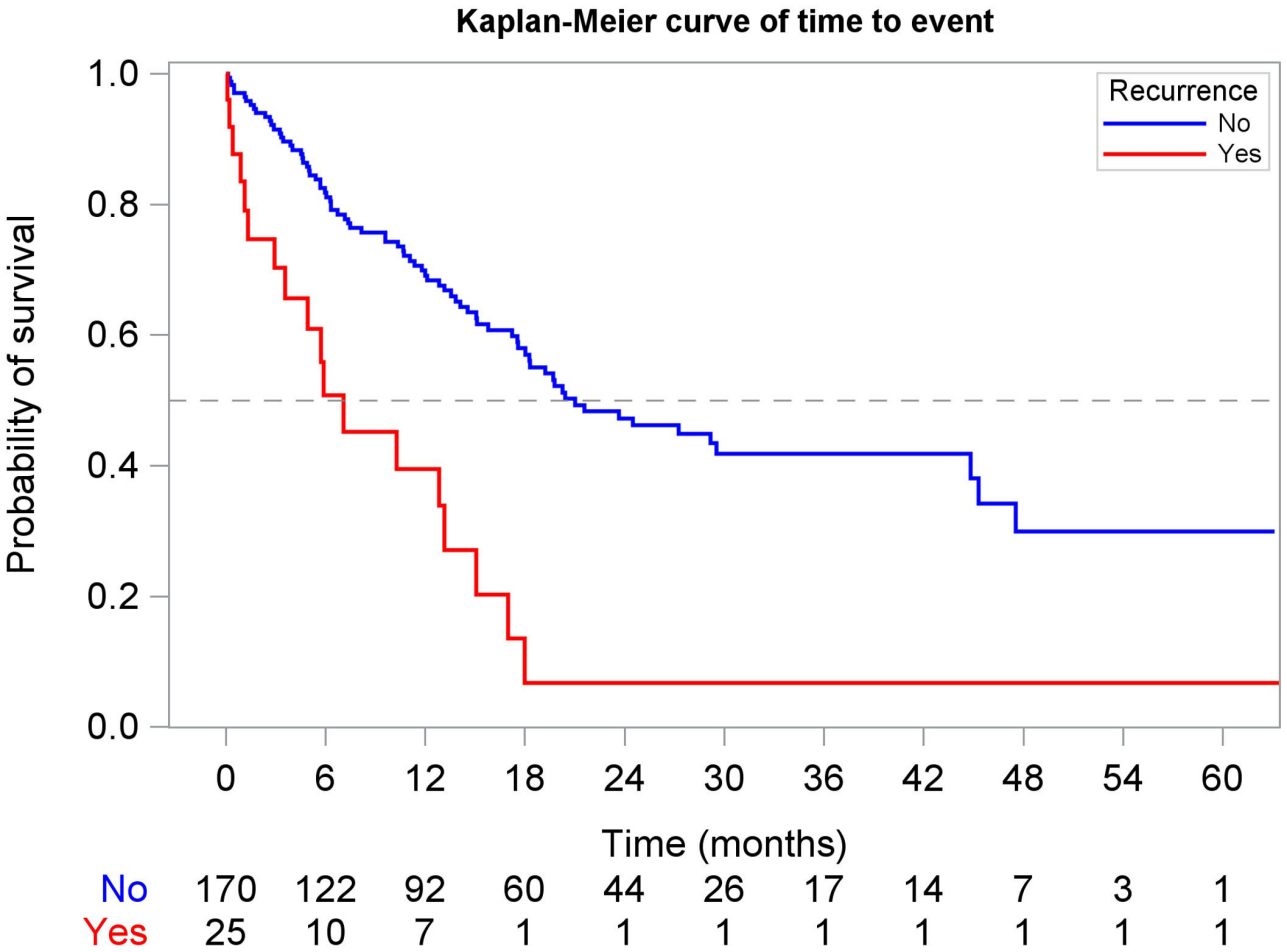
Landmark Kaplan-Meier curve of rwOS at 6 months, stratified by recurrence status.

**Figure 7:**
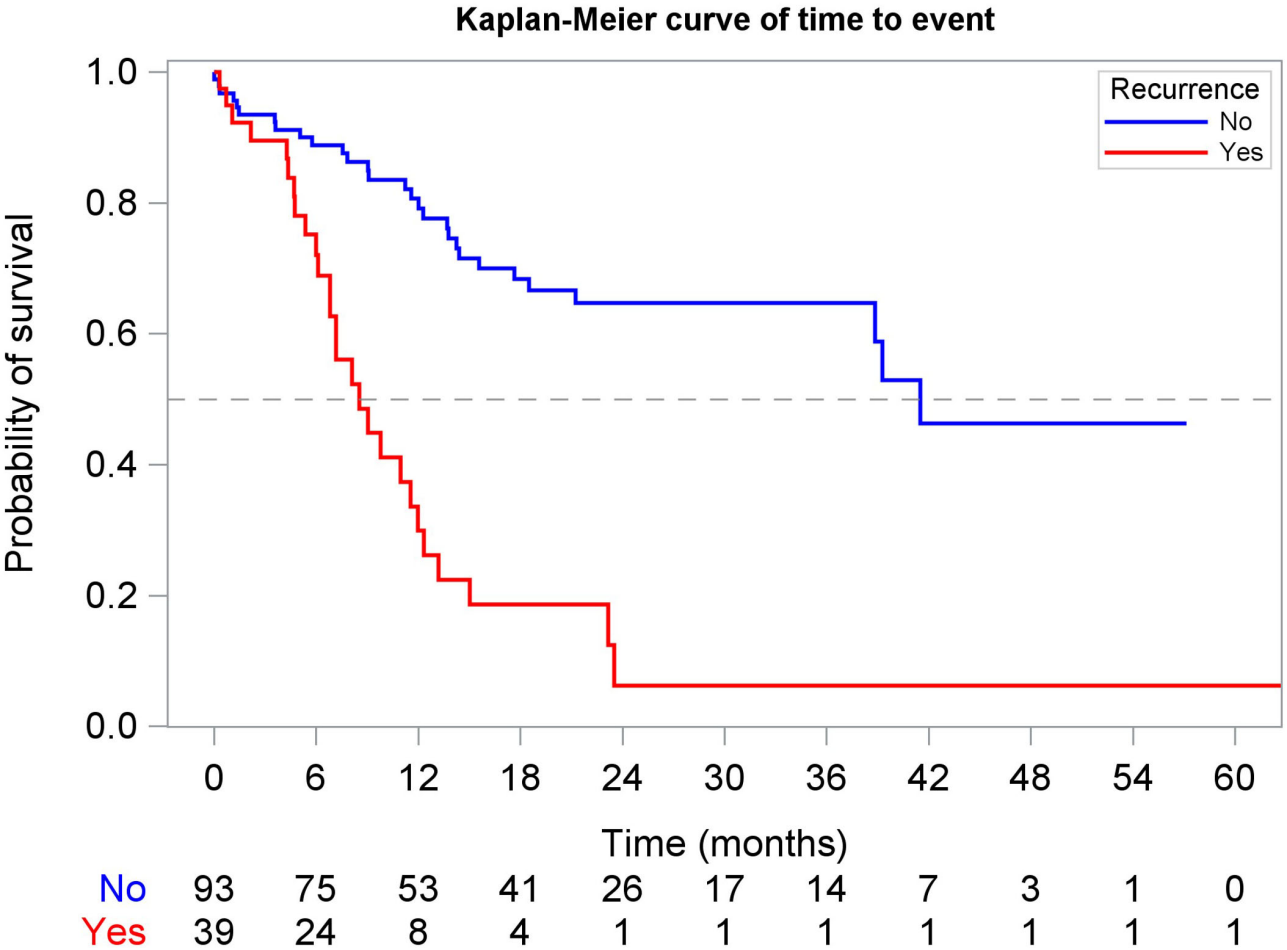
Landmark Kaplan-Meier curve of rwOS at 12 months, stratified by recurrence status.

Landmark analyses revealed that at 6 months (**Figure 6**), eligible patients who experienced recurrence had a median rwOS of 7.1 months (95% CI: 2.9, 13.2) compared to 21.0 months (95% CI: 17.6, 44.8) for those without recurrence (adjusted HR: 2.87, 95% CI: 1.51, 5.45). At 12 months among available patients (**Figure 7**), the rwOS for the recurrence group was 8.5 months (95% CI: 6.8, 12.0) compared to 41.5 months (95% CI: 38.8, NR) for the no recurrence group (adjusted HR: 4.15, 95% CI: 2.10, 8.21). At 18 months among available patients, the recurrence group had a median rwOS of 5.6 months (95% CI: 3.1, 9.0) compared to a non-arrived median rwOS (95% CI: 32.8, NR) for the no recurrence group (adjusted HR: 5.73, 95% CI: 2.28, 14.35). Overall, patients who experienced recurrence were 5.85 times greater risk of death compared to those who did not experience recurrence during the study period (adjusted HR: 5.85, 95% CI: 4.08, 8.39) from a time-dependent Cox model.

**Figure 8:**
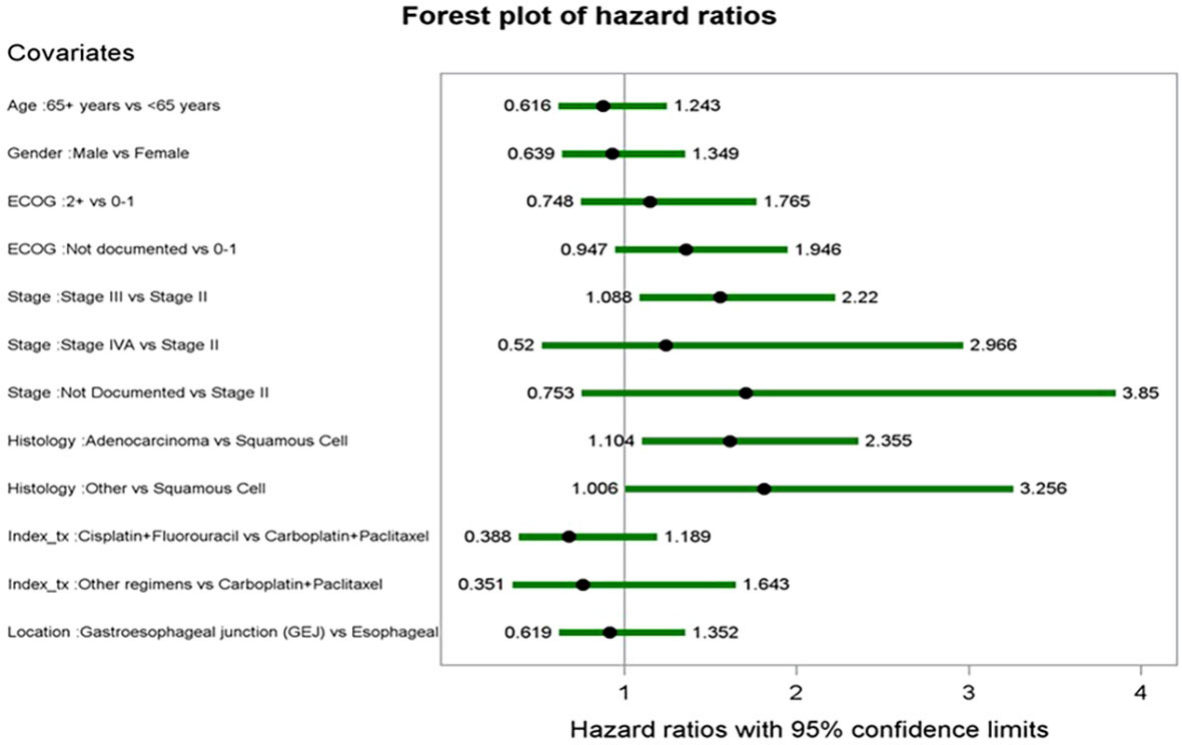
Forest plot of hazard ratios from multivariate cox proportional hazards model for rwOS.

Covariates and their corresponding HRs from the multivariable Cox proportional hazards model for rwOS are featured in **Figure 8**. Factors associated with reduced rwOS included adenocarcinoma (vs. squamous cell) with an HR of 1.61 (p < 0.0281). Factors in the final model but not statistically significant included age group at index, gender, ECOG at baseline, histology, index regimen, and tumor location.

**Figure 9:**
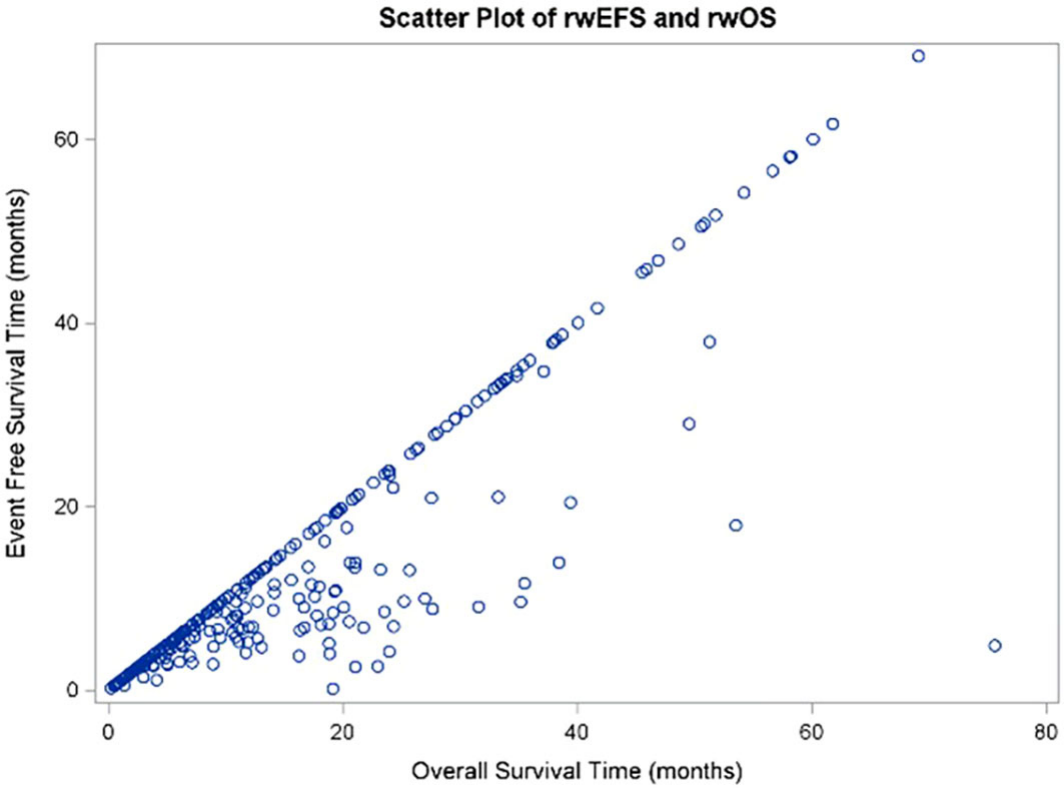
Scatter plot of rwEFS and rwOS correlation using Kendall's Tau correlation coefficient.

Finally, the results of the correlation analysis evaluating the relationship between rwEFS and rwOS indicated a strong correlation (r = 0.8; 95% CI: 0.8, 0.9; **Figure 9**) between these two endpoints.

## 4 Discussion

The results of this study provide insights into the real-world characteristics, treatment patterns, and outcomes of patients with locally advanced EC/GEJC adenocarcinoma treated with dCRT between 2015 and 2021 in a community-based oncology care setting. Unlike other real-world studies that have used non-US data sources or administrative claims data restricted to a single insurance type, this research leveraged a large, nationally representative EHR database from community oncology clinics (15–17). The primary objective was to address a significant evidence gap in the current literature concerning the real-world experience of US EC/GEJC patients treated with dCRT who are not eligible for surgery. Because of the higher risk of recurrent disease in this population, this study had a particular focus on comparing patients who did and did not experience recurrence following treatment.

Overall, the study cohort was largely representative of the US EC/GEJC population. The study population had a median age of 73 years, which is slightly older than the median age of 68 for esophageal cancer patients in the US (18). Despite this age difference, the majority of the patients were men, a large proportion were White/Caucasian, and most had adenocarcinoma histology, which aligns with the expected national demographics for this population (3). The study population in this analysis included a more diverse patient population than the RTOG 85–01 clinical trial; the RTOG 85–01 trial population included a younger study population with only 26% of patients aged ≥70 years (19).

Treatment patterns for the study population also aligned with clinical care standards. The majority of patients in this analysis received carboplatin and paclitaxel, consistent with clinical guidelines (20). Among those with documented radiation dosing, over half received radiation dosages between 50 and 50.4 Gy. A real-world study which analyzed registry data from the Netherlands had similar findings, with the majority of CRT patients receiving radiation doses of 50.4 Gy (90%) and carboplatin plus paclitaxel (97%) (21). In this analysis, only a small proportion of patients initiated LOT2, largely due to high mortality rates after LOT1; these findings are similar to other real-world treatment pattern studies of patients with EC/GEJC where less than half of patients continued on to a later line of therapy (22, 23). However, treatment options remain limited, underscoring the challenge faced by patients with EC/GEJC.

Progressive or recurrent clinical events were common in this study cohort. Two-thirds of the patients experienced a clinical event, such as locoregional or distant metastasis (recurrence or progression) or death, with a median rwEFS of 8.9 months (95% CI: 7.7, 10.6). This median rwEFS is shorter compared to another real-world study conducted in a university setting in Germany, which reported a median rwEFS of 18.3 months for neoadjuvant chemoradiotherapy (nCRT) and 12.7 months for dCRT (24). The difference in outcomes may be attributed to the smaller sample size, a slightly younger patient population with a median age of 65 (nCRT, n=40) and 68 (dCRT, n=55), and the prevalence of squamous cell histology in the other real-world study, in contrast to the adenocarcinoma histology in this study (24).

As anticipated for a population with high rates of progression or recurrence, survival outcomes were poor, especially for those who experience a recurrence during their treatment regimen (25). For rwOS in this study, over half of the patients died before the end of the study follow-up, with a median rwOS of 18 months. Median rwOS is comparable to another real-world study including study population (n=157) with a similar age and sex distribution from a tertiary referral center, which reported a median rwOS of 22.9 months (95% CI: 18.0, 27.9) (26). Additionally, the landmark analysis revealed that at 6, 12, and 18-month timepoints, patients who experienced recurrence had shorter rwOS compared to those who did not experience recurrence. Furthermore, patients who did not experience recurrence during the study period demonstrated a survival benefit. This trend is consistent with another real-world study, which reported a 5-year rwOS rate of 62.1% for patients with 1-year progression-free survival (PFS) and 83.7% for those with 2-year PFS (27). Another study assessing patients with esophageal cancer who developed recurrence found a 3-year rwOS rate of 39.5% compared to a 3-year post-recurrence survival rate of 22.6% (28).

Moreover, rwEFS and rwOS were estimated to be strongly correlated (r = 0.8; 95% CI: 0.8, 0.9) in this analysis, demonstrating that a delay in recurrence is associated with improved survival in this patient population. In a meta-analysis of 11 published randomized controlled trials (RCTs) of dCRT interventions in unresectable locally advanced EC, EFS and OS were also estimated to have strong correlation with a slope coefficient of 0.91 (p < 0.001) and R^2^ of 0.80 (95% CI: 0.38, 0.96) (29). Similarly, a meta-analysis of 26 trials with patients with operable locally advanced EC/GEJC demonstrated strong correlation between disease-free survival (time to recurrence or death) with Kendall’s Tau coefficients ranging from 0.73-0.87, stratified by treatment type (surgery, chemotherapy followed by surgery, or dCRT followed by surgery) (30). Overall, this analysis highlights potential greater OS benefit that can be achieved by preventing or slowing disease recurrence.

In addition to these results underscoring the significant survival benefit for patients who did not experience recurrence, these analyses support the use of EFS as a validated surrogate endpoint for OS. The development of new treatments can be very time-intensive, with clinical trials often spanning several years, in part because of the amount of follow-up time required to evaluate OS (31). However, there is evidence to support endpoints like EFS as a predictor of long-term clinical benefit in a clinical trial setting, which may help reduce these delays. In EC/GEJC patient populations characterized by high recurrence rates, the adoption of surrogate endpoints may expedite regulatory pathways for novel investigational therapies that demonstrate early benefit in EFS outcomes.

Patients in this analysis with a recurrence event generally had shorter survival outcomes, despite follow-up time being longer in the recurrence subgroup than the non-recurrence subgroup, suggesting there may be diminishing treatment efficacy for second-line therapies after recurrence. In this study, rwOS was slightly longer in the landmark survival at 6, 12, and 18 months for patients without recurrence likely driven by diminished treatment efficacy after recurrence. Other real-world studies have noted similar findings for patients who experience recurrence or progress during treatment (25, 31). Additionally, survival bias in the recurrence group may contribute to longer follow-up time, where patients must live long enough to experience a recurrence to be present in that subgroup. However, the landmark OS analysis was adjusted for time with the same starting point for both recurrence and non-recurrence subgroups. Furthermore, there may be a higher proportion of missing data in the non-recurrence stratification, where patients are being censored earlier than those with a recurrence.

There are some inherent strengths and limitations associated with the underlying data that should be considered when interpreting the results of this study. Like all retrospective studies utilizing real-world data sources, the data analyzed for this manuscript were not collected for research purposes. As such, there may be inconsistencies in reporting practices across oncology centers. Further, there may be some misclassification bias present in the data due to errors recorded at the site of care. However, this bias may be mitigated given the availability of chart abstraction and medical review as well as standard quality assurance measures performed for the iKM EHR data. Patients who received care outside of The US Oncology Network, including those treated at academic hospitals are not reflected in this study. Despite this limitation, insights into routine clinical practice at community-based oncology centers can still be drawn from this data source. Some variables were omitted from this analysis given higher rates of missing data (e.g., specific biomarkers). Additionally, the presence of missing data for baseline characteristics (e.g., ECOG, race, smoking status, etc.) may also limit generalizability; although even within stratifications, missing data was never higher than 20% when present.

Generally, earlier diagnosis and treatments that delay recurrence in EC/GEJC may lead to better patient outcomes and quality of life improvements for patients not eligible for surgery. Moreover, the results of the landmark and correlation analyses, indicate that a delay in recurrence is associated with improved survival. The findings of this analysis highlight a gap in effective treatments options and can provide real-world data insights regarding patient characteristics, treatment patterns, and clinical outcomes for patients with locally advanced, unresectable EC/GEJC treated with dCRT at community-based oncology centers. Further, these results suggest EFS is an adequate surrogate endpoint for OS. In conclusion, the results of this study underscore the need for the development and uptake of more effective therapies for EC/GEJC patients to improve survival outcomes.

## Data Availability

The data analyzed in this study is subject to the following licenses/restrictions: The health data used to support the findings of this study are restricted by The US Oncology Research Institutional Review Board in order to protect patient privacy. For this reason, data used to support the findings of this study have not been made available. Requests to access these datasets should be directed to https://www.ontada.com/.
